# Electrospun Medical Sutures for Wound Healing: A Review

**DOI:** 10.3390/polym14091637

**Published:** 2022-04-19

**Authors:** Lin Xu, Yanan Liu, Wenhui Zhou, Dengguang Yu

**Affiliations:** 1 School of Materials and Chemistry, University of Shanghai for Science and Technology, Shanghai 200093, China; 212203153@st.usst.edu.cn (L.X.); 1935022506@st.usst.edu.cn (W.Z.); 2 Shanghai Engineering Technology Research Center for High-Performance Medical Device Materials, Shanghai 200093, China

**Keywords:** sutures, wound healing, electrospinning, nanofibers, drug delivery, medical polymers

## Abstract

With the increasing demand for wound healing around the world, the level of medical equipment is also increasing, but sutures are still the preferred medical equipment for medical personnel to solve wound closures. Compared with the traditional sutures, the nanofiber sutures produced by combining the preparation technology of drug-eluting sutures have greatly improved both mechanical properties and biological properties. Electrospinning technology has attracted more attention as one of the most convenient and simple methods for preparing functional nanofibers and the related sutures. This review firstly discusses the structural classification of sutures and the performance analysis affecting the manufacture and use of sutures, followed by the discussion and classification of electrospinning technology, and then summarizes the relevant research on absorbable and non-absorbable sutures. Finally, several common polymers and biologically active substances used in creating sutures are concluded, the related applications of sutures are discussed, and the future prospects of electrospinning sutures are suggested.

## 1. Introduction

Medical sutures refer to special medical threads used in surgery to stop bleeding, which can hold the surrounding tissues of the wound together or squeeze blood vessels to achieve hemostasis [1]. For soft tissues such as skin, muscles, tendons and ligaments, the wound repair device used needs to be highly elastic and flexible. The properties of the ideal sutures include: (1) It can maintain sufficient strength during the wound healing process, and should also be able to elongate to adapt to wound dropsy, and retract back to the original length with the wound retraction; (2) after the wound is healed, it can be degraded and absorbed by itself, leaving no foreign body; (3) no inflammation; (4) no irritation and carcinogenicity; (5) easy to dye, sterilize, disinfect and other treatment; (6) can form a safe and firm knot; (7) easy to make, low price, and can be produced on a large scale [2,3].

The increasing incidence of Surgical Site Infections (SSI) due to wound infections has led to increased treatment costs, increased hospitalization rates, longer duration of treatment, severe morbidity and high mortality [4,5]. Surgical sutures are the implantation of foreign bodies in the patient’s body, which inevitably causes tissue reactions that may lead to inflammation and other complications [6]. The source is the presence of microorganisms in the wound to form bacterial biofilm. Bacterial biofilm is a sticky membrane layer on the surface of bacteria, which is over-accumulated by a large number of bacteria and surrounded by secreted fibrin to form a collective community. Such biofilms are often found on non-living surfaces, such as hospital walls, medical devices, and implants, as well as biological surfaces, such as surgical sites, wounds and other tissue sites. There are bacterial biofilms that protect the growth of bacteria that can lead to chronic wound infection [7,8]. Chronic wound infection further aggravates the complexity of wound treatment that the use of conventional antibiotics cannot satisfy [9,10]. Methicillin-resistant *Staphylococcus aureus* (MRSA) has been reported to have caused 20,000 related deaths in the United States in 2017 alone and remains one of the important causes of infection-related deaths [11]. Common species in hospitals include gram-positive (*Staphylococcus epidermidis* (*S. epidermidis*) and *Staphylococcus aureus* (*S. aureus*)) and gram-negative (*Pseudomonas aeruginosa* (*P. aeruginosa*) and *Escherichia coli* (*E. coli*)), and it is highly desired to develop surgical sutures with excellent performance and effective antibacterial and anti-inflammatory properties. Nanofibers can play their important roles in developing new kinds of sutures to replace the traditional threads in terms of properties and functional performances such as drug delivery and wound healing [12].

At present, the method of preparing nanofibers has been phase separation [13], self-assembly [14] and electrospinning, of which electrospinning is recognized as an effective method for the preparation of nanofibers through electrohydrodynamic atomization procedures [15,16]. Nanofiber membranes prepared by electrospinning have a series of well-known advantages, such as high specific surface area, high surface volume ratio and high porosity, and their structure is similar to the human extracellular matrix, which can be effectively exploited to promote cell adhesion, proliferation, migration and differentiation [17,18,19]. During the preparation of electrospun nanofibers, it is convenient to encapsulate some bioactive ingredients such as growth factors, inorganic nanoparticles, antibacterial drugs and herbal extracts to promote wound healing [20,21,22,23,24,25,26,27,28,29]. Therefore, it is widely used not only as a polymer processing technology, but also as a facile approach to develop novel functional nanomaterials [30,31,32,33,34]. Meanwhile, the nanofiber properties can be easily manipulated by changing the relevant process parameters during the manufacturing process or using solvents with different properties [35,36,37].

**Figure 1:**
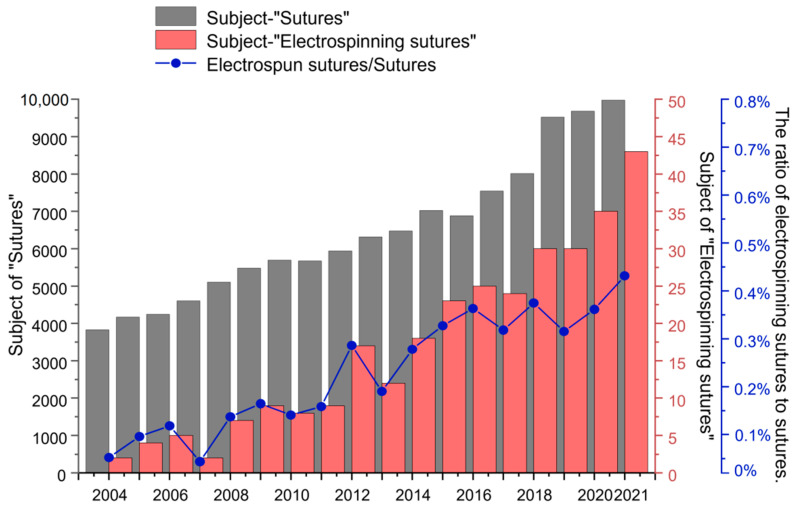
Statistics on the number of publications retrieved on the “Web of science” with the subject of “Sutures” and the subject of “Electrospun sutures”. The line chart represents the ratio of electrospinning sutures to sutures.

In “Web of Science”, the search results for “Sutures” and “Electrospinning sutures” are shown in Figure 1. It can be observed that from 2004 to 2021, “Sutures” have been a research hot spot, and the number of articles published is always increasing. Nearly 300 articles are searched with the subject of “Electrospinning sutures” during the last decade. At the same time, the proportion of the number of electrospun suture articles to the number of suture articles is also elevating year by year. These data demonstrate that there is still a lot of research space for the preparation of sutures using electrospinning technology, and electrospun nanofibers will develop into a new type of medical suture with wide application prospects. This review first introduces the structure and performance analysis of sutures, then classifies sutures and summarizes the polymer materials used in common sutures, followed by the methods of preparing sutures, which are mainly based on electrospinning technology, and finally comment on the future prospects for electrospun nanofiber-based sutures.

## 2. Structure of Sutures

**Figure 2:**
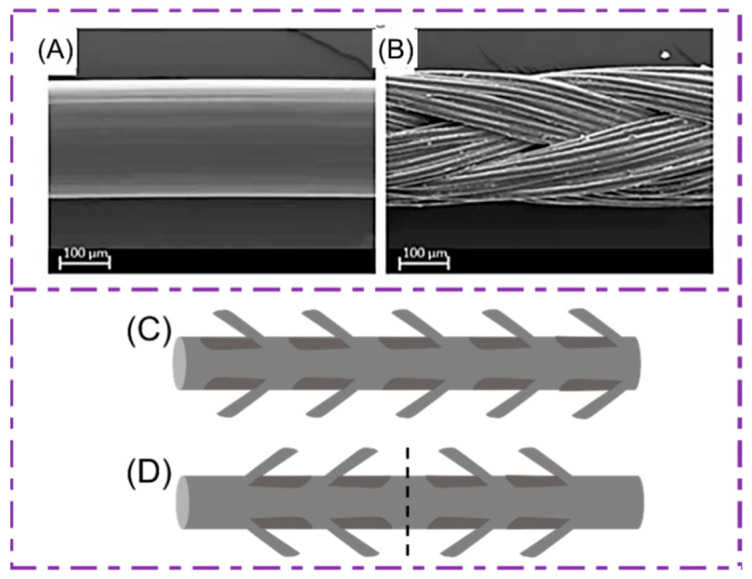
(**A**) Monofilament and (**B**) multifilament sutures. Reprinted from Ref. [38]. (**C**) Unidirectional barb sutures; barbs all point to the same direction (**D**) with two-way barbs stitched together, and the two sets of barbs are pointed in opposite directions according to the midpoint of the length of the sutures. Reprinted with permission from Ref. [39] Copyright 2021 John Wiley and Sons.

Traditionally, the sutures can be composed of the monofilament sutures (Figure 2A) or the multifilament sutures (Figure 2B). The monofilament sutures are single strand structures, while the multifilament sutures are woven from multi-stranded fibers.

### 2.1. Monofilament Sutures

Monofilament suture is a single strand structure with a small microbial contact area, which can effectively reduce the possibility of bacterial growth. When using a monofilament suture to close a wound, multiple knots are required. In addition, only a low knotting force can be used, otherwise it is easy to break. Its advantages are that the surface is smooth and relatively easy to be knotted. Moreover, there is less resistance when passing through the tissue, which is less damaged to the tissue. Therefore, monofilament sutures are suitable for suturing contaminated wounds [39].

### 2.2. Multifilament Sutures

Multifilament sutures are woven together by multiple strands of threads. Braided sutures have greater flexibility and better tensile strength than non-woven sutures, but the latter have less tissue response and scar formation [6]. They are usually coated, and the suture nodules are highly firm but not suitable for usage in treating infected wounds. Multifilament sutures are not only stronger, but also have good operability, and multi-filament sutures produce better knots than monofilament sutures. Because of its special structure, less knotting is required. However, the multi-strand structure of braided sutures is susceptible to infection. Because the structure has small gaps, it can provide a place for bacteria to grow and reproduce. Moreover, compared with monofilament sutures, the structure of multifilament sutures with more small gaps allows more fluid to pass through the sutures, which is more likely to cause tissue inflammation at the wound site. A larger inhibition band can be observed when the multifilament sutures wrap the active pharmaceutical ingredient (API), such as silver or antibiotics; this indicates that the multi-gap structure of the multifilament suture plays an important role in drug delivery. The braided structure of the multifilament suture allows for a greater chance of surface coating, allowing more APIs to be adhered to than the monofilament suture. Therefore, multifilament sutures have better structural flexibility and provide greater odds for adding APIs. This makes the multifilament sutures coated with APIs have good physiological activity (such as antibacterial, anti-inflammatory, antioxidant, etc.) [38,39,40,41].

### 2.3. Barb Sutures

Barbed sutures are surgical sutures with barbs on the surface of the sutures to penetrate the tissue, completing wound closure without knotting the sutures. They include bi-directional or one-way/unidirectional knotless surgical sutures. The monofilament sutures are improved to make barbed sutures. The one-way barb sutures have barbs in the same direction and the needle is pressed against one end (Figure 2C). Another type of barbed suture modified from monofilament sutures is held in place with an anchor or knot to prevent the barbs from moving in the opposite direction, which are similar to one-way barb sutures. The two sets of barbs of this suture press the midpoint on both sides of the suture opposite each other, pressing the needle on both ends for suture anchoring, and do not need a knotted circle to fix it, and is suitable for wounds that are easily separated on both sides (Figure 2D).

Compared with traditional monofilament and multifilament sutures, barb sutures are untangled sutures. They can be absorbable or non-absorbable sutures, containing specially designed barbs that can invade tissue and fix them. Unidirectional barbed sutures eliminate the need for knotted sutures, greatly increasing the tensile strength of the sutures while also reducing tissue response. Thus, barb sutures are considered an alternative to all soft tissue closures for traditional sutures [6].

In addition, barb sutures reduce the probability of bacteria present on the sutures, thus avoiding inflammation and other complications in wounds. Although barb sutures are widely used in clinical surgery, the barb tip design of the sutures may inadvertently puncture the surgical glove, causing the infection to metastasize to the surgeon and lead to further infection of the patient’s wound [42]. In addition, a cut-type barb may weaken the seam core, thinning the diameter of the seam and reducing tensile strength. However, if handled carefully, barbed sutures can exert good antibacterial activity to ensure the wound healing of skin tissue [43].

## 3. Performance Analysis of Sutures

According to studies [44,45], the reasons for the failure of wound healing caused by surgical sutures have the following causes: the fracture of the suture line, the weak knot of the suture, and the frictional damage between the suture and the tissue. Therefore, the performance study of surgical sutures has mainly focused on its physical properties. Among them, the low tensile properties of surgical sutures can lead to secondary cracking of the wound, the low friction properties can lead to difficult knotting and the high relaxation properties will lead to reduced suture support strength during postoperative healing [46]. Three sutures commonly used in surgery were studied and analyzed. The parameters include the tensile and relaxation properties before and after the knotting, the simulated surgical knotting, the influence of sutures at different speeds, different loads and different surface morphologies and structures on their frictional properties, which are investigated for providing basic data for the design optimization of sutures and surgical suture knotting operations [47]. In recent years, there have been fewer reviews of suture performance studies [48,49,50]; therefore, this review will further summarize the physical properties of sutures and provide reference for future research.

### 3.1. Physical Performance Analysis

During the weeks or months of wound healing, the strength of tissue at the wound site increases, even closer to that of the tissue before the injury. The most basic principle for selecting sutures is to use as thin and tensile sutures as possible with minimal response to tissue [51]. As concluded by Huang in Table 1, the sutures of different diameters and the difference between the diameters of adjacent sutures are listed.

The above table is used to identify the suture diameter of the suture line according to the specifications of the United States Pharmacopeia (USP), where each specification corresponds to the size of its stitching, and the medical staff can select the suture required according to the actual needs.

**Figure 3:**
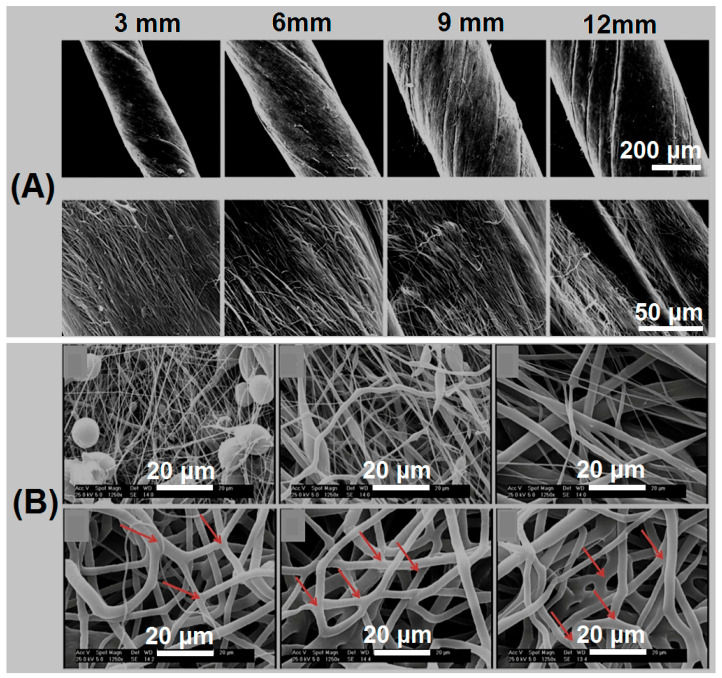
(**A**) SEM images of sutures made from core sheath nanofiber films with widths of 3, 6, 9 and 12 mm. Reprinted with permission from Ref. [55] Copyright 2017 Elsevier; (**B**) SEM images of PCL scaffolds with different tensile strengths (0.5 mpa, 0.8 mpa, 1.0 mpa, 1.3 mpa, 2.3 mpa and 3.0 mpa); arrows show fusion and bonding of the adjacent fibers. Reprinted with permission from ref. [56] Copyright 2017 Elsevier.

The tensile strength of the suture line is closely related to its size, and some scholars have conducted relevant studies on the relationship between the diameter of the suture line and the tensile properties. Nout et al. studied that as the diameter of the suture material increases, the creep of the material decreases. Under different loads, the creep of the polyoxycyclohexanone suture line is smaller, while the creep of the polypropylene suture line is slightly more increased than the former. Therefore, the former is more suitable for closing the wound when a lot of force is required to approach the edge of the two wounds [53]. Tobias et al. selected phosphate buffer saline (PBS) as a solvent protection solution [54]. They soaked the synthetic absorbable suture material in it, and compared the tensile properties before and after soaking. The results demonstrated that the difference in tensile strength and maximum elongation was respectively as high as 63.6% and 34.2% for different sutures of the same size. Compared to several commercial sutures currently in clinical use, Maxon sutures have the highest tensile strength and elongation at break. Chen et al. prepared gentamicin/polyoxyethylene F127-silver Polycaprolactone(PCL) nuclear sheath nanofiber strips by electrospinning, which were further rotated into medical sutures [55]. The mechanical properties of nanofiber strip sutures with different widths were compared. Their scanning electron microscope (SEM) images are shown in Figure 3A, and the authors further verified that the tensile strength of the nanofiber bands with widths of 9mm and 12mm was significantly higher than that of the nanofiber bands of 3mm and 6mm. In general, the fiber size will inevitably increase whether the tensile strength of the suture line is increased. On the contrary, when fibers receive different tensile strengths, the morphology of fibers will also be changed accordingly. Asvar et al. studied the mechanical properties of PCL fiber scaffolds [56]. As shown in Figure 3B, the SEM images display the PCL fibers with different tensile strengths. When the tensile strength reaches 0.8 MPa, the fiber morphology begins to straighten, but beaded and worm-like fibers are still present. For PCL fibers that can withstand tensile strength of 1.3 MPa, it is not difficult to find from the arrow pointing that there is no fusion between adjacent fibers and no beading. As tensile strength continues to increase, so does the fiber diameter.

In a word, the physical and mechanical properties of sutures should be fully considered in the preparation of sutures. This allows sutures to be used safely, such as when they are knotted to close wounds.

### 3.2. Operational Performance Analysis

Usually when a wound is sutured with sutures, there is friction not only between the sutures and the sutures, but also between the sutures and the wound tissue. If the friction coefficient is too large, it will lead to the fracture of the suture line or tissue damage, causing wound infection and preventing healing. An earlier study demonstrated that reducing the coefficient of friction between the suture and the skin could make the knot easy to slide to control the lashing tension, making the overall knot less secure [57]. Up to date, there are very limited studies to disclose the influence of the morphological structure of the suture line on the coefficient of friction. Zhang et al. studied the surface morphologies of three different structural sutures: multifilament sutures (silk), monofilament sutures (propylene) and coated multifilament sutures (vicryl), and the results demonstrated that sutures with monofilament structures or coatings can effectively reduce the coefficient of friction at the interface with the tissue [58]. Bezwada et al. studied the monofilament sutures of polypropylene (poliglecaprone 25), demonstrating that they exhibited minimal resistance and excellent tensile properties when passing through the tissues relative to composite braided sutures [59].

To reduce the surface friction of the suture and improve the efficiency of surgery, many researchers apply lubricant or wax to the surface of the suture to achieve this goal. Viju et al. discussed the effect of chitosan coating on the frictional properties of suture lines, and the results demonstrated that not only the toughness and tensile strength of the suture lines increased with the increase in chitosan concentration, but the chitosan-coated sutures had excellent antibacterial activity against both *S. aureus* and *E. coli* [60]. Griesser et al. created a thin polymer coating on the suture line by cosmeticizing the sutures. The frictional properties of the coated suture line were evaluated by measuring the dynamic friction between the suture line and the myocardium of the sheep, and it was found that the coating effectively avoided the sticky slip behavior of the suture line [61]. 

### 3.3. Biological Performance Analysis

**Figure 4:**
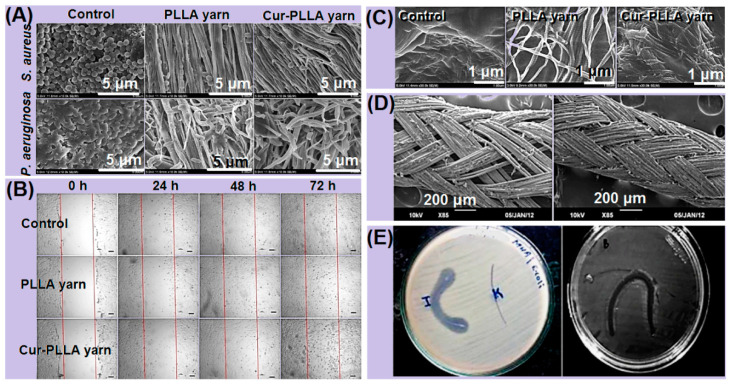
(**A**) Commercial suture (Vicryl (3-0) Ethicon), pure PLLA sutures and curcumin-laden PLLA sutures of SEM images of the inhibition of *S. aureus* and *P. aeruginosa*. (**B**) Comparison chart of the number of promoted cell migrations in the control group and sample group at 0, 24, 48 and 72 h. (**C**) Comparison figure of collagen fiber deposition on the surface of the control group and sample group. Reprinted with permission from Ref. [62] Copyright 2021 Elsevier. (**D**) SEM micrograph of the surface of the biodegradable suture of the nano-silver coating. (**E**) Nano-silver particles; antibacterial figure of nano-silver-coated biodegradable sutures (labeled I) and uncoated sutures (labeled K) inhibit the region of *E. coli* and *S. aureus*. Reprinted with permission from Ref. [63] Copyright 2021 Springer Nature.

For the inhibition of postoperative wound infection at the surgical site, antibacterial and anti-inflammatory drugs, such as curcumin, gentamicin and nano-silver, are added to the suture material. Performance evaluations of these sutures are usually conducted, such as in vitro antibacterial assays, in vitro drug release assays and cell migration assays. Richard [62] et al. detected the adhesion degree of *S. aureus* and *P.* *aeruginosa* on the surface of the control group and the pure Poly(l-lactic acid) (PLLA) nanowire and PLLA nanowire loaded with curcumin. As can be observed from Figure 4A, with the attachment of curcumin, the adherence of the two strains gradually decreased from complete coverage at the beginning, which minimizes the inhibitory effect of bacteria transmitted in the form of thin films on wound healing. Meanwhile, Figure 4B showed the difference of the cell number in the treated region between the sample and the control group at 0, 24, 48 and 72 h, respectively. The sutures loaded with curcumin demonstrated better cell migration at the wound site. It is clear from Figure 4C that the sutures loaded with curcumin produced a large amount of collagen fiber deposition on the surface of the wound during the remodeling stage. These results are sufficient to prove that it was a suitable material for antibacterial sutures. Another study aimed at coating the surface of the woven suture with nano-silver particles (Figure 4D) [63], and its inhibitory effect on the infection at the surgical site was assessed through antibacterial experiments. As shown in Figure 4E, the suture with a nano-silver particle coating has a pronounced antibacterial inhibition zone.

## 4. Electrospinning Processes

**Figure 5:**
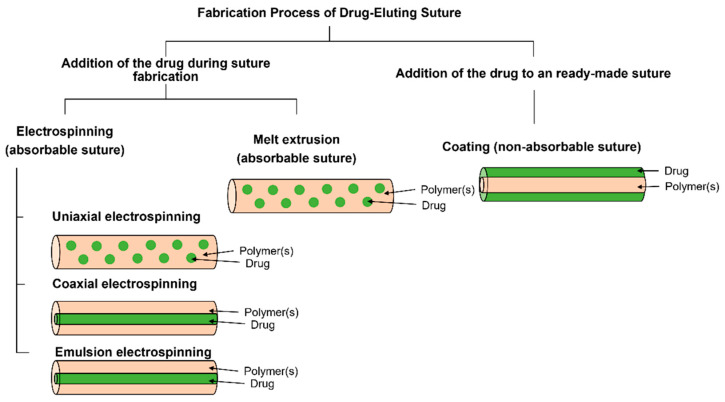
The manufacturing process of drug-elution sutures, where electrospinning and melt extrusion are mainly used to produce absorbable drug-eluting sutures, while coating technology is mainly used to manufacture non-absorbable drug-eluting sutures. Reprinted with permission from Ref. [39] Copyright 2021 John Wiley and Sons.

Current manufacturing techniques for the production of pharmaceutical elution sutures include electrospinning, melt extrusion and coating (Figure 5). As an indispensable medical device for hospital surgery to treat wounds, surgical sutures can effectively improve the healing rates of wounds and reduce the pain of patients. Electrospinning is widely used to prepare nanofibers, which can provide the sustained release of antibiotics, anticancer drugs, proteins, DNA and RNA, living cells and a variety of other growth factors [64,65]. The loading of targeted drugs for specific treatments is an advantage of electrospinning [66,67,68], which has attracted increasing attention from several overlapped scientific fields.

**Figure 6:**
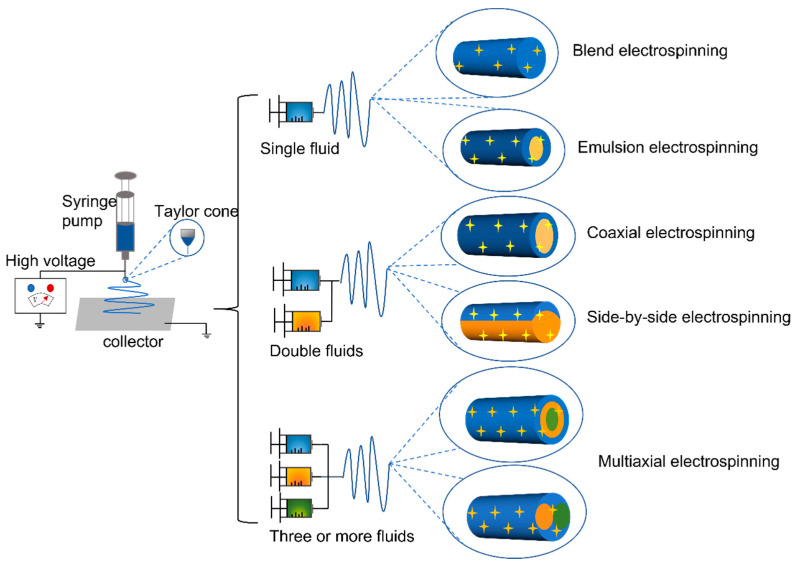
Classification of electrospinning processes.

Electrospinning equipment mainly includes four parts: (1) a high-pressure generator, (2) one or several syringe pumps, (3) a spinneret, and (4) a collector [69]. The above parts are organized together to manipulate the interactions between the electrostatic energy and the working fluids. Its working principle is that under a constant high-voltage power field, the syringe equipped with a polymer solution is placed in the field, and the nozzle of the syringe is subject to the action of the high-voltage electric field, pushing the solution to be continuously spun. During the working process, when the applied high voltage is enough to overcome the surface tension of the solution at the nozzle, the so-called “Taylor cone” will be formed at the tip of the needle, and the voltage will be continuously increased. When the electric field is large enough, the solution can be sprayed in the form of a trickle, and the solution continues to evaporate the solvents and solidify during the spraying process. Finally, a nonwoven fabric-like fibrous web is deposited on the collector [70]. The concept of electrospinning was proposed as early as 400 years ago, evolved from the original electrospray technology, and has developed to the present. It is classified from its fluid strands: single-fluid electrospinning (blended electrospinning and emulsion electrospinning), double-fluids electrospinning (coaxial electrospinning and side-by-side electrospinning) and multi-fluids electrospinning (tri-fluid tri-layer coaxial electrospinning and other multi-fluid electrospinning) (Figure 6) [71].

### 4.1. Single-Fluid Electrospinning

Single fluid spinning includes blend electrospinning, emulsion electrospinning and suspension electrospinning. Among them, blend electrospinning is the most traditional method to prepare nanofibers. Blend electrospinning nanofiber technology is the process of dissolving the polymer used in a suitable solvent to make a spinning fluid. It is optional to add suitable bioactive substances to give them special functions. This technology has been widely used in the following fields, sewage treatment [72], drug sustained release [73,74,75], wound dressings [76,77], food packaging [78,79], sensors [80] and so on. Solid nanoparticles were loaded into polymer spinning solutions so that nanoparticles were present on or inside the final nanofibers [81,82,83]. For example, Rasekh et al. incorporated SiO_2_ nanoparticles into a polymer solution for electrospinning to make hydrophobic membranes for wastewater purification [84]. However, blend electrospinning technology involves the solubility of polymers and bioactive substances in solvents, and too low dissolution will lead to uneven composition of fibrous tissues, affecting the performance of use. 

For the disadvantage of mixed electrospinning, emulsion electrospinning successfully utilizes a two-phase dispersion system of oil-in-water type. The polymer organic solvent in the mixed emulsion can evaporate and cure to form a protective layer, and the interior is a drug dispersion phase. It is made of typical nuclear shell structure nanofibers, which effectively avoids the problem of polymer insoluble in blended yarn solution. At the same time, the loading of biologically active substances is realized, which is widely used in the field of the controlled release of drugs [85,86].

### 4.2. Double-Fluid Electrospinning

Different from the single-fluid electrospinning technology, the spinneret of the double-fluid electrospinning adopts a double-layer composite nested structure. There is a certain gap between the inner layer and the outer layer so that the polymer solution flows smoothly. Finally, the core-sheath structure nanofibers are obtained [87,88]. Among the many electrospinning technologies, coaxial electrospinning is widely used to prepare core-sheath structure nanofibers [89]. Using this preparation method, the position of the bioactive substance in the medical suture nanofibers can be changed, thereby changing its performance. For example, adding the bioactive substance to the core solution can realize the nanofiber control drug to release slowly and prolong the treatment time of the suture to the wound. If a biologically active substance is added to the sheath solution, the drug can be directly in contact with the wound and achieve a wound treatment effect in a short period of time. With coaxial spinning we can control the flow rate of the sheath fluid and indirectly control the amount of drug released [90]. Therefore, the successful application of coaxial electrospinning technology has made a breakthrough contribution to the field of the controlled release of drugs, and also provides the possibility for the preparation of medical sutures for drug loads. He et al. studied the development of antimicrobial elution sutures by mixing electrospinning and coaxial electrospinning of PLLA with tetracycline hydrochloride (TCH), successfully combining electrospinning with aligned fiber collection, and providing a reference for the manufacture of pharmaceutically loaded medical sutures by electrospinning technology [91]. 

With further research on coaxial electrospinning technology, in 2010, Yu et al. reported electrospinning experiments using pure solvents (non-spinning liquids) as sheath working fluids [92]. For any functional raw material that cannot be film-formed, this modified coaxial electrospinning technology can prepare it into nanofibers, that is, the two layers of the core sheath solution do not have to be polymers. It greatly expands the raw material selection of nanofibers, further enhancing their functionality and applications [88]. Therefore, with this new type of coaxial electrospinning technology, we can choose a wider range of raw materials when preparing medical sutures, and make them into a variety of functional nanofibers to promote wound healing [93,94].

### 4.3. Multi-Fluid Electrospinning

Multi-fluid electrospinning technology is a further improvement of dual-fluid electrospinning technology. It changes the spinneret structure and increases the number of fluids. For example, it is designed as a three-layer or multi-layer spinneret structure. This spinning technology with more fluid passages can be combined with polymers and bioactive substances of different properties. This leads to the development of nanofibers with higher performance and more complex structures. In the field of medical sutures, we can achieve the long-lasting release of drugs in the sutures at the wound, and even achieve antibacterial, scar elimination and biodegradation effects at the wound simultaneously. It has broad research prospects.

### 4.4. In Situ Electrospinning

Among the many electrospinning technologies, in situ electrospinning came into being in order to use medical materials that are convenient and can be customized to fit wounds. Although electrospinning technology has been widely used in the preparation of wound dressings, it is all used in in vitro treatment, and there was a recent study of depositing nanofibers directly on living organs to achieve wound hemostasis. Zhang et al. used the long-needle electrospinning method in combination with minimally invasive surgery to embed the long needle into the laparoscopic tube [95]. The electrospun nanofibers could be manufactured by laparoscopy and deposited directly onto living organs. It demonstrates the advantages of quickly stopping bleeding compared with traditional hemostasis methods, less postoperative inflammatory response and faster recovery.

### 4.5. Improved Receiver Used to Prepare Nanowires

Electrospinning is an efficient and easy method for preparing nanofibers. The presence of a receiver in electrospinning equipment is critical. It determines the structure, distribution state, productivity and even performance of nanofibers. More commonly used are flat plate receivers, which collect nanofibers only on one side of the collector. To circumvent some of the limitations of the flat plate collector, researchers have developed an in-house rotating edge-sharpened disc collector, producing highly aligned nanofibers and bundled nanofiber yarns as surgical sutures [96]. Instead, a stainless-steel disc receiver can be used, which rotates at a certain speed, applying a voltage that causes the polymer solution to deposit on the edge of the disc, and then manually removing the fiber bundle [97]. More people use funnel-shaped rotators combined with positive and negative voltages, plus a winding mechanism with a certain speed, to make highly aligned nanofiber wires [98]. A new study has developed a 3D hydrogel collector in the shape of ear cartilage that can be moved to completely cover the nanofibers and reproduce the collector’s felt, providing a more effective reference for the preparation of nanofibers in various fields [99].

## 5. The Type of Sutures and the Polymer Materials of Sutures

**Figure 7:**
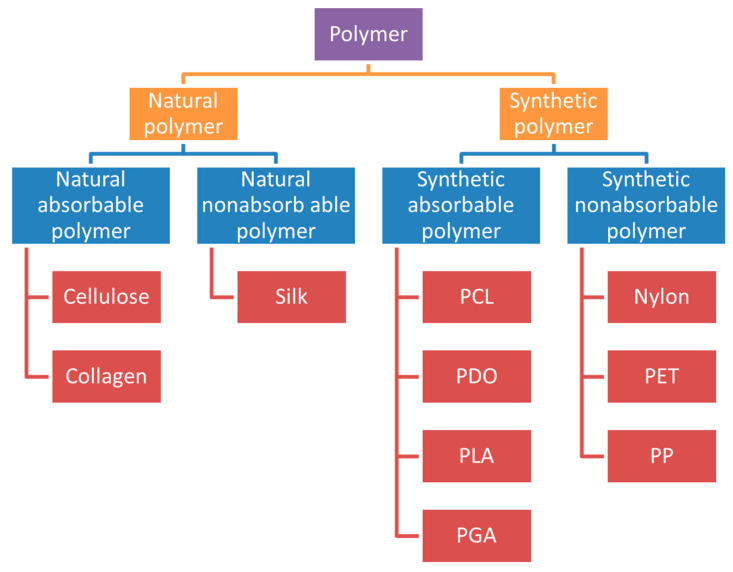
Common polymers used in medical sutures.

The different types of sutures summarized in this article are absorbable sutures and non-absorbable sutures. With the development of sutures, it is possible to develop absorbable smart sutures and sutures that are antibacterial and capable of removing scars. According to the needs of different types of wounds, different medical sutures can be selected. At the same time, the choice of suture material is mainly based on the tissue to be repaired. As surgical procedures and treated tissues vary, it is important to understand the basic types of suture materials [100]. The common polymers used in medical sutures are included in Figure 7. 

Commercial sutures currently in use are Dexon (monofilament/braided sutures made of polyglycolic acid(PGA)), Vicryl (Braided sutures made of polylactosin 910), Maxon (monofilament sutures made of polygluconate), Quill™ SRS (Barbed sutures made of poly-p-dioxanone (PDO)), Ethibond (woven sutures made of polypropylene(PP)), MaxBraid (braided sutures made of polyethylene(PE)), Monosof (monofilament sutures made of nylon) and so on [42].

### 5.1. Absorbable Sutures 

Absorbable sutures are presently a hot topic; they are easy to degrade in the body or even be completely absorbed, and it is important to avoid postoperative wound infection and reduce patient pain. Absorbable sutures have been proven to lose 50 percent of their tensile strength in tissues within 60 days, resulting in degradation [42]. Among them, natural absorbable sutures degrade due to proteolysis, while synthetic absorbable sutures degrade due to hydrolysis [101]. Absorbable suture materials are divided into natural absorbable suture materials and synthetic absorbable suture materials. At present, the commonly used absorbable suture materials are sheep catgut, cellulose, polycaprolactone (PCL), poly-p-dioxanone (PDO), polylactic acid (PLA), poly (lactic-co-glycolic acid) (PLGA), polyglycolic acid (PGA), polyurethane (PU) and so on (Table 2).

Catgut as a biological suture thread was originally made from the intestines of sheep or cattle, dried and then medically treated. It is a monofilament, absorbable suture with excellent processing function that degrades naturally in the human body through the action of proteolytic enzymes [102]. Later, it developed into a thin layer of chromium compound on all sides of the catgut, which became a chrome catgut. It can be engulfed by macrophages in tissues such as other heterogeneous proteins, and the sutures can generally be maintained for 7 to 10 days. If absorption is faster within an infected wound, the chrome catgut can be applied to all subcutaneous tissues for pulling and suturing, except for excessive inter-wound tension or inflammation and infection wounds [103].

Oxidative regenerated cellulose (ORC) can be used as a hemostatic material, but the current ORC medical suture has not really achieved clinical application. Li et al. prepared novel woven suture threads by OXY-regenerated cellulose (TORC) mediated by TEMPO (2,2,6,6-tetramethylpiperidine oxide) to achieve sewable material with biodegradable properties and ideal mechanical properties [104]. With the prolongation of oxidation duration, the carboxyl content in the TORC suture line gradually increased from 5.1% to 10.4%. The oxidation reaction also gradually reduced the strength, weight and diameter of the suture line, promoting degradation. Overall, the development of this suture reveals potential prospects for clinical applications. Prior to this, Wu et al. studied bacterial cellulose nanocrystals (BCNCCs) and regenerated chitin (RC) fibers to form BCNC/RC sutures. This suture exhibits good biodegradability and no cytotoxicity. In addition, it can promote cell proliferation, which is conducive to wound healing [105].

Based on the above two naturally biodegradable polymers, researchers have also been working to synthesize biodegradable polymers. For example, Chen et al. studied the biodegradable properties of monofilament suture made from bacterial bio-polyester poly (3-hydroxybutyrate-co-4-hydroxybutyrate) (P3BV-co-HB) [106]. In the in vitro degradation experiments, its strength hardly changed, and its original fracture strength was 65%, which was comparable to that of the commercially available suture material catgut. In rat implantation experiments, no significant tissue response was shown during degradation in vivo, and the tissue response was milder than that caused by the chrome catgut. The molecular weight change of the suture line in vivo is very similar to the change in in vitro degradation and is ideal for surgical sutures. Compared with P3HB, P4HB has better toughness and is more suitable for avoiding suture fracture problems [107]. Keridou et al. prepared P4HB monofilament fibers by electrospinning. After the test of morphological characterization and physical properties, it was found that P4HB monofilament fiber exhibited good mechanical properties, and the fiber was suitable for cell adhesion and proliferation [108]. In addition, it can be completely absorbed within 12–18 weeks after implantation in the human body, which has good application prospects as an absorbable suture material.

PCL is an aliphatic synthetic biodegradable polyester with good biocompatibility, good organic polymer compatibility and good biodegradability [109]. PCL sutures prepared during electrospinning that control relevant parameters such as polymer concentration, solution feed rate, applied voltage and nozzle-to-collector distance maintain good tensile strength and suture retention [110]. In addition, the combination of PCL and ethyl cellulose (EC) as a new bio-friendly shape memory polymer has development potential in the field of bio-suture line. It not only has good biocompatibility and biodegradability, but also has excellent mechanical properties and shape memory performance. Shape memory temperature can be adjusted while maintaining the tensile strength of the suture [111].

PDO is a colorless, biodegradable polyester with excellent biocompatibility, biodegradability and mechanical flexibility for use in medical devices, tissue engineering scaffolds and controlled drug delivery [112,113]. Mixing semi-crystalline PDO and Poly(l-lactide-co-ε-caprolactone) (PLACL) in suture composition will enhance toughness while also enabling customized degradation times [114]. As a commonly used monofilament suture material, PDO can still maintain a tensile strength of 55%-70% for 3-4 weeks, which helps to prolong the wound support time [115]. Zhu [116] et al. successfully prepared super-strong nano-composite fiber felts based on biodegradable PDO and chitin nanocrystals (ChiNCCs) by electrospinning.

PLA sutures are widely used as absorbable sutures in modern medical surgery, and its degradability has always been a problem for people to use this type of suture. Liu [117] et al., in order to control the degradation cycle of PLA sutures, recombined carbon nanotubes (CNTs) with PLA sutures and tracked and analyzed the structure and properties of sutures in the degradation process. The results demonstrated that the strength effective time of the original PLA sutures was 13.5 weeks. Comparing with this strength effective time, that of the CNTs/PLA sutures was increased to 26.6 weeks, effectively extending the time of their strength during degradation.

PLGA is a synthetically biodegradable polymer used to make absorbable sutures. Its degradation rate can be customized to suit its application, making it a potentially excellent controlled release conveyor. It can be degraded in vivo into two harmless products (lactic acid and glycolic acid) [118]. PCL and PLGA mixed polymers are prepared into suture fiber scaffolds. The tensile strength of these sutures is significantly higher than that of pure polymers to prepare suture lines, and the porosity and cell permeability are also optimized to a certain extent [119]. PGA, like PLGA, has long been included by the U.S. Food and Drug Administration (FDA) as a biodegradable polymer material and is widely used in biomedical fields.

**Table 2:** Commonly used absorbable suture materials.

	Name	Structure	Characteristic	Preparation Method	Ref.
Natural absorbable suture material	Catgut/chrome gut	Monofilament	Monofilament, easy to degrade, low tensile strength, susceptible to bacterial infection	Washing and drying	[102]
	Regenerated cellulose	Monofilament	Ideal biodegradability and mechanical properties	Wet spinning	[104]
Synthetic absorbable suture material	P3BV-co-HB	Monofilament	Strong toughness, biodegradable, non-toxic, promote cell proliferation	Blend electrospinning	[106,108]
	PCL	Monofilament or woven	Good biocompatibility, degradability, mechanical properties and shape memory properties are excellent	Blend electrospinning	[110]
	PDO	Monofilament	Colorless, biodegradable, mechanically flexible	Blend electrospinning	[116]
	PLA	Monofilament	Biocompatible, good degradability	Blend electrospinning	[117]
	PLGA	Monofilament	Degradable, non-toxic and harmless, good tensile properties	Blend electrospinning	[118]
	PU	Monofilament	Biocompatible, degradable	Blend electrospinning	[120]

As manufacturing technology continues to evolve, researchers are working on developing new-feature sutures that attempt to combine biodegradable polymer materials with electro-optical capabilities to create absorbable smart sutures. For example, Liu et al. were inspired by the multi-layered structure of the “core-shell” of natural spider silk fibers. A bionic, antibacterial and sensing suture made from regenerated silk fibroin was designed, which has a hierarchical structure and isomeric functionalization [121]. It can reduce inflammation and bacterial infection at the wound site, and measure the tension of tissue and sutures. It helps tissue healing and monitors function in real time by releasing controlled drugs and growth factors. Since then, sutures have not only been able to aggregate and fix damaged tissue, but also are biologically active and electronically/optically capable components. It provides a good research basis for wound healing research. Prior to this, the development of intelligent surgical sutures with excellent mechanical properties and shape memory behavior has been realized. The earliest were Lendlein and Langer Joo, who performed minimally invasive surgery using self-tightening, biodegradable sutures [120]. However, the mechanical strength and structural properties of absorbable polymer sutures are insufficient to meet the basic biochemical mechanical requirements for their normal operation. Joo et al. later utilized biocompatible and biodegradable PU and PCL blends to obtain shape memory properties [122]. High crystalline PCL is used as the hard segment, and the PU synthesized with isosorbide is used as the soft segment. The experiments have proven that 30% PU/PCL mixture has good shape memory characteristics, fixed shape rate of 95%, shape recovery rate of 71%. In addition, the 30% PU/PCL mixture can automatically form knots at 40 °C, demonstrating the potential of intelligent stitching applications. Houshyar et al. developed multifunctional sutures with temperature sensing and infection control, incorporating functionalized nanodiamond (FND) and reduced graphene oxide (rGO) in the biodegradable PCL [123]. The suture is optically verified to have temperature sensing capabilities, while the surface is easy to apply antibiotics to reduce the risk of bacterial infection of the wound.

All in all, there are many materials that can be used to prepare absorbable sutures, which has always been a concern of researchers in the field of medical sutures, and its degradability is also a priority choice for more patients. Combined with more natural degradable substances, the toxicity of the polymer will be better reduced, further improving the practical safety of the suture line.

### 5.2. Non-Absorbable Sutures

Non-absorbable sutures refer to sutures that cannot be absorbed and degraded by the human body after implantation, which can provide long-term support for tissues and require secondary surgical removal. Poor healing and scarring of sutured wounds were observed when non-absorbable sutures were used [124]. Compared with natural suture materials, synthetic suture materials are not only biocompatible, but also have better mechanical properties than natural materials. Common non-absorbable suture materials are silk thread, nylon, polypropylene (PP), polyester (PET) and so on (Table 3).

Silk is a compound filament suture that combines excellent mechanical properties and biocompatibility and is widely used in the biomedical field. However, because its main component is protein, it cannot lose most of its tensile properties in the body within 60 days, and it takes longer to degrade. Therefore, silk is classified as a natural non-absorbable suture line [125]. When silk is used alone, it is usually dyed black with oil, wax or silicone to improve visibility and toughness. Despite the enhancing effect, the tensile strength of the silk suture is lower than that of all currently available sutures [126]. In addition, the braided nature of the silk sutures allows pathogens to enter the wound and have a high probability of infection [127]. This can lead to infection and inflammation in the tissues in which they are placed [128]. Therefore, silk sutures are not responsible for antibacterial action. Without affecting the strength and use of wound healing, it is very important to develop antibacterial filament sutures. Uncoated treatment may affect the physical and operational properties of silk sutures. The preparation and modification of the thread suture material needs to be optimized [129,130].

Nylon is a synthetic, non-absorbable monofilament suture made of chemically inert polyamide polymer fibers with a certain tensile strength, and is a monofilament structure that reduces the reactivity of tissues and the rate of wound contamination. It has an antithrombotic effect, ensures wound closure and is commonly used for surface inflammation and non-inflammatory wound closure of the skin [131]. Polyamide sutures are non-absorbable monofilament sutures with tough fibers that have high tensile strength, elasticity and gloss [132]. Experiments have demonstrated that nylon has excellent tensile properties and is conducive to surgical wound closure, but its elastic modulus is small and easy to return to its original state, resulting in instability of the knot. Compared to PET and polylactosin-coated sutures, the surface colony formation unit level of nylon sutures is significantly lower, and the accumulation of microorganisms in monofilament nylon sutures is lower than that of other woven sutures [133]. In most cases, acute trauma can be sutured with a chrome catgut to suture the subcutaneous layer and a nylon suture to suture the epidermis.

PE is a synthetic, non-absorbable monofilament suture. It exists in the form of a monofilament made from the catalytic polymerization of propylene, which has low tissue reactivity and high tensile strength, similar to nylon [134]. One of its significant advantages is its ease of removal and high plasticity and ability to adapt to wound edema, and it is a suture line with a better skin healing process, because its surface is naturally smooth and easy to implant and remove, maintaining the same tensile strength after implantation. It can also pass through skin tissue and cause minimal inflammatory response; however, a smooth surface may reduce the safety of the knot [135,136].

The so-called non-absorbable PET suture here is an non-absorbable multifilament woven suture consisting of polyethylene terephthalate. These sutures are often coated, such as poly butyrate, PTFE and silicone, which reduce tissue resistance [137]. This type of suture has extremely high tensile strength, only lower than metal, and the loss of tensile strength after suturing is also small or even nothing. In addition, these sutures provide ongoing support for slowly healing tissues and produce minimal tissue response [127].

**Table 3:** Commonly used non-absorbable suture materials.

	Name	Structure	Characteristic	Preparation Method	Ref.
Natural non-absorbable suture material	silk	monofilament	Good biocompatibility, low tensile strength, easy bacterial infection,	Wet spinning	[125]
Synthetic non-absorbable suture material	nylon	monofilament	A certain tensile strength can reduce tissue infection and fight thrombosis	Melting, forming, cooling	[132]
	PP	monofilament	Low tissue reactivity, high tensile strength, high plasticity, adapted to wound edema	Chemical synthesis, usually surface coating to prepare sutures	[135]
	PET	multifilament	Extremely high tensile strength, good operability, not easy to degrade	Chemical synthesis, usually surface coating to prepare sutures	[137]

### 5.3. Bioactive Substances for Medical Sutures

Nanofibers prepared from electrospinning also have a significant advantage in that they can add functional bioactive substances to the polymer. If the experiment only relies on polymer materials to prepare nanofibers, it is not better to achieve antibacterial, promote cell adhesion proliferation and other functions. Therefore, people are more inclined to add some bioactive substances to the polymer solution to achieve specific functional needs. At present, the bioactive substances confirmed in the preparation of medical sutures are: silver nanoparticles, triclosan, nitric oxide (NO), GO, growth factors, curcumin, heparin etc. Infection caused by bacterial attachment to the suture line is a serious obstacle to the use of sutures, and studies have demonstrated that antimicrobial sutures can significantly reduce the incidence of infection at the surgical site [138].

**Figure 8:**
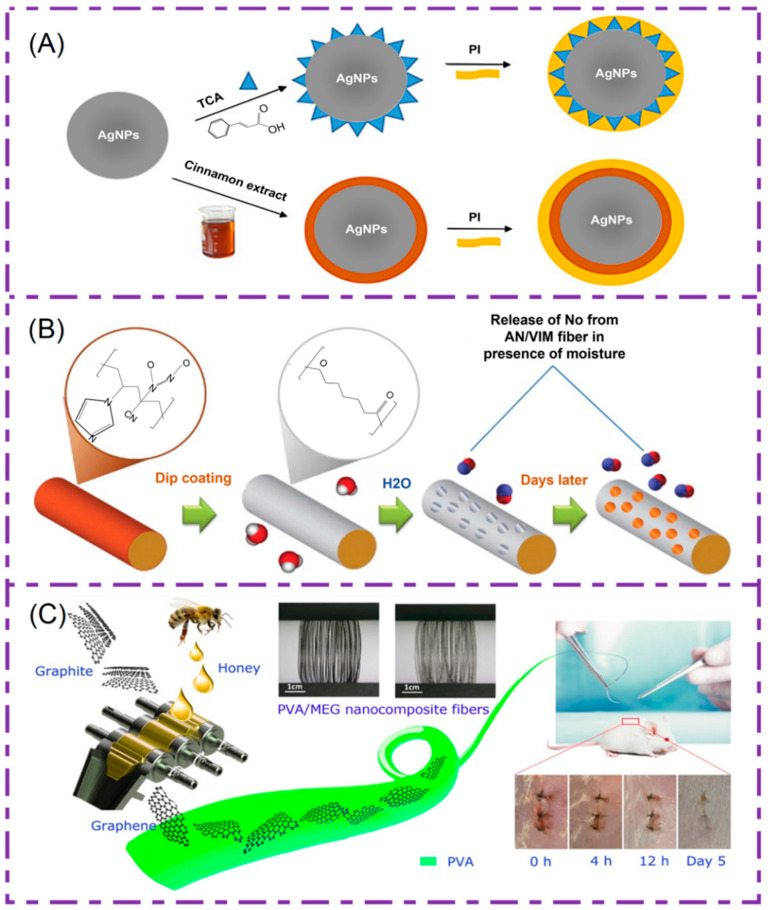
(**A**) AgNPs/TCA/PI nanofibers and AgNPs/Heads/PI schematic diagram of nanofiber structure. Reprinted from Ref. [140]. (**B**) PCL overlaid AN/VIM copolymer fibers NO controlled release. Reprinted with permission from Ref. [142] Copyright 2012 American Chemical Society. (**C**) Preparation of PVA/MEG nano-composite fibers and experimental diagrams of mice in vitro. Reprinted with permission from Ref. [143] Copyright 2018 American Chemical Society.

Nano-silver particles have significant antibacterial properties and are commonly used to be antibacterial on the surface of medical devices. In 2018, Rouhollahi et al. developed PGA-PLGA electrospinning nanofibers containing silver nanoparticles and twisted them into nanofiber yarns [139]. The focus of his research is to explore the antibacterial properties of silver nanoparticles in yarn. In addition, it has been proven through in vitro antibacterial experiments that yarn containing 3% silver nanoparticles has obvious antibacterial effect on gram-positive bacteria and gram-negative bacteria. It can be used as a suitable candidate for antibacterial sutures. Later, Edis et al. combined hydrophobic trans-cinnamic acid (TCA) and natural cinnamon bark extract (Cinn) with nano-silver particles, respectively. Hydrophilic povidone iodine (PI) was then added and biodegradable PGA was dipped to make sutures [140]. The composition of the structure diagram is shown in Figure 8A; this mixed antimicrobial/fungicide/drug delivery system has the advantage of preventing SSI and biofilm formation.

Triclosan inhibits the synthesis of fatty acids in bacteria, acts as an effective fungicide, and prevents bacteria from colonizing, after which, the infection occurs. The first antibacterial surgical suture coated with triclosan (woven polylactose 910) was approved by the U.S. FDA in 2002 [141]. As an antibacterial coating for medical sutures that have been used clinically, it can effectively prevent bacteria from adhering to the surface of the suture thread and avoid wound infection, providing an effective reference for further research.

There are also scholars who have studied the incorporation of NO into surgical sutures. NO is synthesized by nitric oxide synthase in vivo, which is inherently antibacterial, achieving the goal of promoting wound healing. In addition, it can promote vasodilation and prevent platelet aggregation. Therefore, incorporating NO into surgical sutures is a viable method for producing antithrombotic and antibacterial materials. Lowe [142] et al. prepared a surgical suture with high durability and tensile strength using acrylonitrile-co-1-vinylimidazole (AN/VIM) copolymer. In addition, this suture can store and release NO. The structure of suture is shown in Figure 8B. The prepared nanofibers did not change the mechanical properties of the fibers after reacting with NO. However, in order to control the release of NO, it must be possible to achieve a sustained antibacterial effect. Therefore, they immersed the suture thread in a solution of PCL with chloroform, forming a porous coating on the fibers to delay release.

High quality graphene oxide is an ideal nanofiber filler material. It can not only improve the mechanical properties of the fibers, but also has a significant antibacterial effect. Based on this, Ma et al. began the study, which firstly obtained graphene through honey as an exfoliating agent, and then prepared PVA/MEG nano-composite fibers [143]. The preparation schematic is shown in Figure 8C, and experimentally proved that the fiber has good antibacterial properties, low cytotoxicity, and has the potential as a suture line.

**Figure 9:**
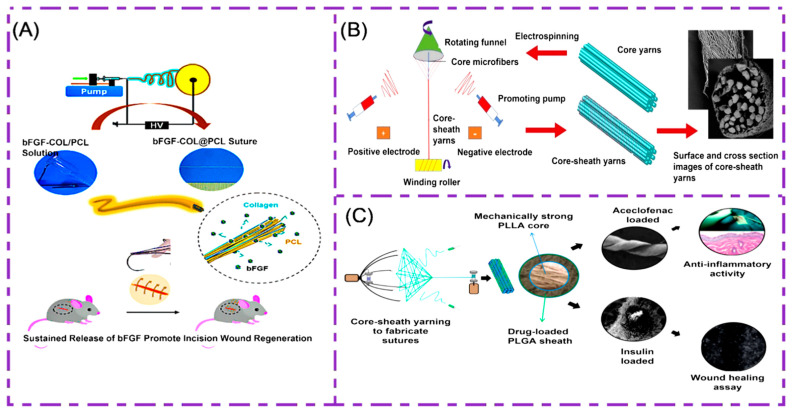
(**A**)**.** Electrospinning preparation of bFGF-COL@PCL suture line principle experimental diagram. Reprinted with permission from Ref. [145] Copyright 2020 American Chemical Society. (**B**) Electrospinning preparation of PLGA core-sheath structure schematic of the suture line. Reprinted with permission from Ref. [146] Copyright 2018 Elsevier. (**C**) PLLA/PLGA/aceclofenac/insulin multifunctional suture preparation. Reprinted with permission from Ref. [96] Copyright 2016 American Chemical Society.

There are many kinds of growth factors, among which the vascular endothelial growth factor (VEGF) can promote cell adhesion and increase in value, thereby promoting the regeneration of new blood vessels. The fibroblast growth factor (BFGF) promotes the formation of new blood vessels and repairs damaged endothelial cells. The transforming growth factor TGF-β has an important regulatory effect on cell growth, differentiation and immune function. Li et al. encapsulate VEGF in regenerated silk fibroin/bladder acellular matrix graft (RSF/BAMG) composite nanofibers by blending and coaxial electrospinning [144], which are determined by in vitro experiments. Nano-sutures containing VEGF are significantly more likely to promote intravascular cell migration and proliferation than those without VEGF, and are promising candidates for medical suture applications. Hu et al. fabricated a multifunctionally aligned electrospinning fiber suture bFGF-COL@PCL [145], as shown in Figure 9A. It is made from biodegradable PCL and collagen (COL) and loaded with bFGF for mechanical strength and controlled drug loading/release. Gu et al. successfully constructed a new type of suture with a “core-sheath” structure by electrospinning equipment [146], as shown in Figure 9B. The suture core structure is ultra-fine PLGA fibers, the sheath is made by electrospinning PLGA and TGF-β1 is loaded to ensure that the suture has a tissue repair function. It has the potential to research and develop multifunctional medical sutures.

As an anticoagulant, heparin is a negatively charged polymer composed of alternating polysaccharide connections. It can effectively stimulate the action of antithrombin, reduce platelet adhesion, and inhibit the formation of blood clots. Previous studies have also reported binding heparin to electrospinning nanofibers as an anionic active ingredient to stimulate the release of cationic growth factors [147]. Based on this, Bae et al. developed electrospinning nanofibers composed of PLGA, polyethylene oxide (PEO) and positively charged copolymer poly(lactide-co-glycolide)-graft-polyethylenimine (PgP). Simultaneous loading of heparin provides a stable release of heparin by interactions between anions and cations. It acts as an anti-thrombotic microvascular suture. [148].

Curcumin is a bioactive substance extracted from turmeric, which has good chemical stability and low toxicity. Combining it with polymers to form nanofibers can show good drug release ability [149,150]. Sharifisamani et al. prepared bilayer nanofibers by electrospinning with a core part made of PCL, the sheath part made of poly(ethylene glycol) (PEG), PLA and PCL, and the drug curcumin was loaded to develop medical sutures for the controlled drug release [151]. The addition of curcumin not only improves the mechanical properties of PCL nanofibers, but also gives the suture antibacterial and healing effect.

Aceclofenac is a new, powerful antipyretic, analgesic and anti-arthritis drug that provides effective anti-inflammatory and analgesic effects in acute wound inflammation. In addition, insulin exhibits a significant pro-cell migration effect. Padmakumar et al. designed the same core sheath structure [96]. As shown in Figure 9C, they used PLLA to make a mechanically strong core, while PLGA was used as a drug-loading shell. At the same time, aceclofenac and insulin act as drugs to enhance the antibacterial properties of sutures and promote wound healing. This further solves the challenge of electrospinning the preparation of multifunctional sutures with high mechanical properties and drug loading.

Viju et al. applied chitosan to woven silk threads to impart antimicrobial properties [152]. Scanning electron microscopy studies revealed the absence and presence of chitosan on the surface of untreated and treated sutures, respectively. The antibacterial properties of chitosan and tetracycline hydrochloride drugs were tested. The combined antibacterial effect of chitosan and TCH drugs is very good and can be used to develop antibacterial sutures to provide protection against microbial infections.

The most important consideration in the selection of polymers and bioactive substances for the preparation of sutures is that the choice of materials must meet the stringent requirements for wound mechanics, reduce wound infections and provide a certain therapeutic effect of the drug [153]. Table 4 lists bioactive substances loaded in nanofiber medical sutures with specific functions.

## 6. Applications

Sutures are probably the most commonly implanted material in the human body and are widely used in all surgical fields. The purpose of suture use is to keep tissues approximate until the wound reaches sufficient tensile strength to prevent cracking during normal physiological activity. The sutured material should cause minimal tissue damage, minimal tissue response, promote primary wound healing and induce minimal scarring [154].

### 6.1. Tendon Rupture Repair

**Figure 10:**
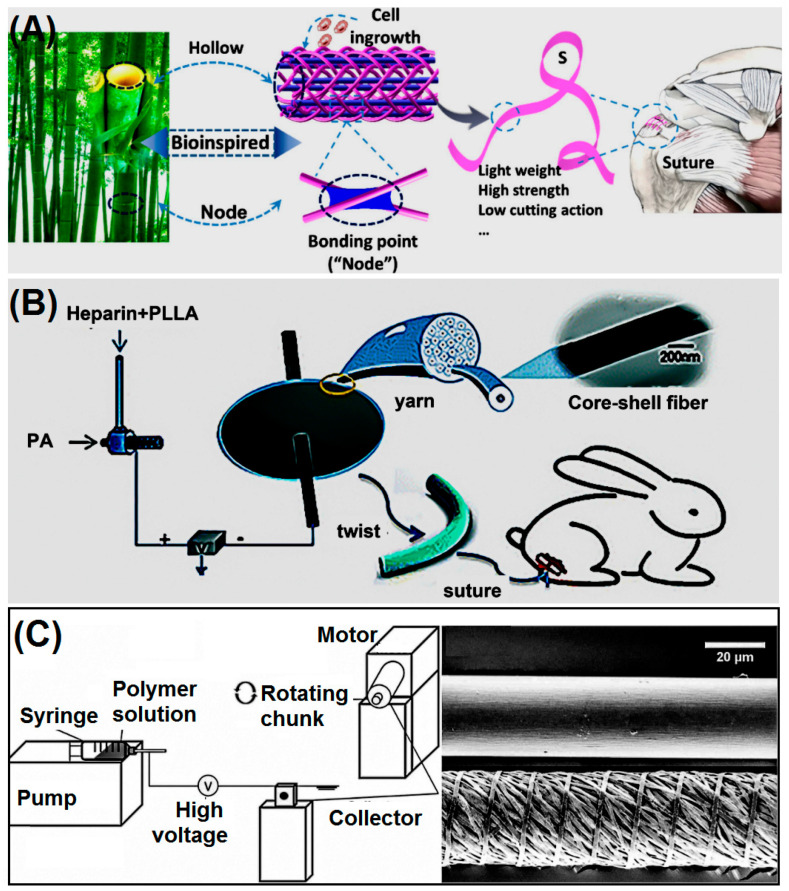
(**A**) Hollow porous suture line structure schematic. Reprinted with permission from Ref. [157] Copyright 2020 Elsevier.(**B**) PLLA/heparin preparation of core sheath structure nanofiber medical suture structure schematic. Reprinted with permission from Ref. [159] Copyright 2021 Royal Society of Chemistry. (**C**) PCL Schematic diagram of the manufacturing system of composite wire sutures. Reprinted from Ref. [163].

Healing of tendon ruptures is a major challenge for musculoskeletal injuries, and sutures play an extremely integral role in tendon repair to reduce pain and restore motor function [155,156]. As shown in the Figure 10A below, Zhang et al. designed a hollow, porous, lightweight tape suture with a controllable structure for tendon repair [157]. Because of the unique structure of the suture line, the suture force pulled out through the tendon is greater. The distance between cutting tendons is smaller, and the suture has been experimentally proved to be cytocompatible and hemocompatible. There is great potential for application in tendon repair. Seo et al. prepared monofilament and multifilament collagen-hyaluronic acid (HA) sutures. Among them, the monofilament is dried on the surface of the suture line coated withCOL-HA. In addition, the multifilament suture is made by electrospinning technology [158]. Comparing the effects of monofilament and composite filament sutures on angiogenesis, cell migration and collagen synthesis in the initial stage of achilles tendon reconstruction in rabbit models, the results demonstrated that COL-HA enhances the migration of new blood vessels and cells in the body. To improve blood supply and promote tendon metabolism and nutrient absorption, Figure 10B, Ye et al. developed a heparin-loaded core-sheath structure nanofiber that exhibited better tendon healing than commercial sutures [159].

### 6.2. Oral and Periodontal Surgery

The oral cavity contains a very diverse microbiome, with more than 700 species reported. Chronic periodontitis (CP) is the most common of oral diseases with bacterial etiology [160]. Most alveolar surgeries require the use of surgical sutures, and the placement and removal of sutures increases the risk of postoperative infection and bacteremia. Meghil et al. used a novel quaternary ammonium compound K21 with antibacterial effects to coat chrome catgut, polyester sutures, silk and nylon sutures, respectively [161]. However, polyester sutures appear to be more effective at lower K21 concentrations, possibly because of the increased absorbance of K21. This study has therapeutic implications for preventing postoperative wound infections.

### 6.3. Prevention of Corneal Repair Infection

Medical sutures are also often used in ophthalmic surgery, and it is estimated that more than 12 million sutures are used annually in eye surgeries, such as corneal transplantation, glaucoma, retinal detachment, vitrectomy and cataract surgery [162]. For 20% to 50% of post-corneal transplantation infections that cause complications, nylon sutures are often used to suture eye wounds. Based on this, it is conceivable to develop anti-inflammatory drugs that maintain sufficient mechanical strength and can load ocular anti-inflammatory drugs on the suture lines to avoid postoperative complications. Parikh et al. prepared monofilament PCL nanofibers by the electrospinning technique [163]. These nanofibers were then twisted into individual sutures (Figure 10C), and they could continuously increase the breaking strength of the suture, while maintaining the tensile strength specified by the USP. At the same time, more small molecule drugs are loaded on the suture, demonstrating a pronounced antibacterial effect on *S. aureus*, reducing the risk of infection.

## 7. Summary and Future Outlook

The role of sutures should not be confined to solely wound closure, but should also promote an effective wound healing in the shortest possible time, prevent infection and alleviate the pain of patients. Improving the mechanical and biological properties of the suture depends to a large extent on the properties of the polymer material used in the preparation of the suture. People are more inclined to use biodegradable synthetic polymer materials, which can not only achieve wound healing and self-degradation, but also avoid patient suffering. In the manufacturing technology of drug-eluting sutures, electrospinning technology is a good choice in order to achieve sutures loaded with a variety of drug active ingredients, and to continuously release drugs during the treatment of wounds to achieve antibacterial and healing effects.

With the continuous development of electrospinning technology, there will be more and more higher performance nanofibers in the future to promote the medical use of medical sutures. However, it has the disadvantage of the inability to produce, and there are still challenges in the mass production of medical sutures through electrospinning technology.

The prevention of the occurrence of postoperative infection and other complications is always a key element to be considered in developing new types of sutures. Today, with the rapid development of the current intelligent manufacturing technology, the combinations of medical sutures with sensing technology to develop intelligent sutures will become a major breakthrough for future smart sutures. Those smart sutures will not only automatically tighten tissues without human intervention to heal wounds, but also will provide real-time monitoring during the healing process for more precise treatment results.

## Figures and Tables

**Table 1:** The difference between the width of the wire diameter range of each specification suture and the adjacent coarse gauge sutures diameter [52].

Specification	Nonabsorbable Surgical Sutures/mm		Absorbable Surgical Sutures/mm					
			Class I		Class II—Single Strand		Class II—Many Strands	
	Width of Sutures Diameter Range	Diameter Difference of Adjacent Coarse Gauge Sutures	Width of Sutures Diameter Range	Diameter Difference of Adjacent Coarse Gauge Sutures	Width of Sutures Diameter Range	Diameter Difference of Adjacent Coarse Gauge Sutures	Width of Sutures Diameter Range	Diameter Difference of Adjacent Coarse Gauge Sutures
12-0	0.008	0.009	——	——	0.008	0.009	——	——
11-0	0.009	0.010	——	——	0.009	0.010	——	——
10-0	0.009	0.010	——	——	0.009	0.010	——	——
9-0	0.009	0.010	0.009	0.010	0.009	0.010	——	——
8-0	0.009	0.010	0.019	0.020	0.009	0.010	——	——
7-0	0.019	0.020	0.029	0.030	0.019	0.010	0.044	0.045
6-0	0.029	0.030	0.049	0.050	0.029	0.030	0.054	0.055
5-0	0.049	0.050	0.049	0.050	0.049	0.050	0.049	0.050
4-0	0.049	0.050	0.049	0.050	0.049	0.050	0.049	0.050
4-0/T	——	——	0.049	0.050	——	——	——	——
3-0	0.049	0.050	0.049	0.050	0.049	0.050	0.089	0.090
2-0/T	0.049	0.050	——	——	0.049	0.050	——	——
2-0	0.049	0.050	0.079	0.080	0.049	0.050	0.059	0.060
0	0.049	0.050	0.069	0.070	0.049	0.050	0.099	0.010
1	0.099	0.100	0.099	0.100	0.099	0.100	0.070	0.071
2	0.099	0.100	0.099	0.100	0.099	0.100	0.039	——
3	0.099	0.100	0.099	0.100	0.099	0.100	——	——
4			0.099	0.100			——	——
5	0.099	0.100	——	——	0.099	——	——	——
6	0.099	0.100	——	——	——	——	——	——
7	0.099	0.100	——	——	——	——	——	——
8	0.099	0.100	——	——	——	——	——	——
9	0.099	0.100	——	——	——	——	——	——
10	0.099	——	——	——	——	——	——	——

Note: “——”represents that this specification data is not available.

**Table 4:** Bioactive substances in nanofiber medical sutures.

Bioactive Substances	Polymers	Characteristics	Preparation Method	Ref.
Silver nanoparticles	PGA-PLGA	Significant antibacterial effect, biocompatibility and degradability	Blend electrospinning	[139]
Triclosan	Polylactose 910	Effective antibacterial avoidance of wound infection	Coating	[141]
NO	Acrylonitrile-co-1-vinylimidazole (AN/VIM).	Maintain good mechanical properties, antibacterial and promote healing	Melt spinning	[142]
GO	PVA	Good antibacterial properties, low cytotoxicity	Blend electrospinning	[143]
Growth factor (VEGF/bFGF/TGF-β)	RSF/BAMG, PCL/collagen, PLGA	Promote cell adhesion and value-add, promote the regeneration of new blood vessels	Coaxial electrospinning	[144,145,146]
Curcumin	PEG, PLA and PCL	Good chemical stability, low toxicity, antibacterial and healing	Blend electrospinning	[151]
heparin	PLGA, PEO and PgP	Reduces platelet adhesion, anti-thrombosis	Blend electrospinning	[148]
Aceclofenac/insulin	PLLA/PLGA	Promote epidermal hyperplasia, cell adhesion migration	Blend electrospinning	[96]
Chitosan/tetracycline hydrochloride	Silk	Antibacterial, bleeding	Blend electrospinning	[152]

## Data Availability

The data supporting the findings of this manuscript are available from the corresponding authors upon reasonable request.

## Abbreviations

SSI	Surgical Site Infections
*S. epidermidis*	*Staphylococcus epidermidis*
*S. aureus*	*Staphylococcus aureus*
*P. aeruginosa*	*Pseudomonas aeruginosa*
*E. coli*	*Escherichia coli*
API	Active Pharmaceutical Ingredient
USP	United States Pharmacopeia
PBS	phosphate buffer saline
PCL	Polycaprolactone
SEM	Scanning electron microscope
PLLA	Poly(l-lactic acid)
PGA	Polyglycolic acid
PP	Polypropylene
PE	Polyethylene
PET	Polyester
PDO	Poly-p-dioxanone
TCH	Tetracycline hydrochloride
ORC	Oxidative regenerated cellulose
BCNCCs	Bacterial cellulose nanocrystals
RC	Regenerated chitin
P3BV-co-HB	Poly (3-hydroxybutyrate-co-4-hydroxybutyrate)
EC	Ethyl cellulose
PLACL	Poly(l-lactide-co-ε-caprolactone)
PLGA	Poly (lactic-co-glycolic acid)
FDA	Food and Drug Administration
NO	Nitric oxide
GO	Graphene oxide
VEGF	Vascular endothelial growth factor
BFGF	Fibroblast growth factor
PEO	Poly(ethylene oxide)
COL-HA	Collagen-hyaluronic acid
